# Variational deep embedding-based active learning for the diagnosis of pneumonia

**DOI:** 10.3389/fnbot.2022.1059739

**Published:** 2022-11-25

**Authors:** Jian Huang, Wen Ding, Jiarun Zhang, Zhao Li, Ting Shu, Pekka Kuosmanen, Guanqun Zhou, Chuan Zhou, Gang Yu

**Affiliations:** ^1^Department of Data and Information, The Children's Hospital, Zhejiang University School of Medicine, Hangzhou, China; ^2^Sino-Finland Joint AI Laboratory for Child Health of Zhejiang Province, Hangzhou, China; ^3^National Clinical Research Center for Child Health, Hangzhou, China; ^4^Department of Research and Education, The Children's Hospital, Zhejiang University School of Medicine, Hangzhou, China; ^5^Department of Computer Science and Engineering, University of California, San Diego, San Diego, CA, United States; ^6^College of Computer Science and Technology, Zhejiang University, Hangzhou, China; ^7^National Institute of Hospital Administration, National Health Commission, Beijing, China; ^8^Avaintec Oy, Helsinki, Finland; ^9^JancsiTech, Hangzhou, China; ^10^Academy of Mathematics and Systems Science, Chinese Academy of Sciences, Beijing, China

**Keywords:** pneumonia diagnosis, active learning, variational autoencoders, brain-like computing, human-centric computing

## Abstract

Machine learning works similar to the way humans train their brains. In general, previous experiences prepared the brain by firing specific nerve cells in the brain and increasing the weight of the links between them. Machine learning also completes the classification task by constantly changing the weights in the model through training on the training set. It can conduct a much more significant amount of training and achieve higher recognition accuracy in specific fields than the human brain. In this paper, we proposed an active learning framework called variational deep embedding-based active learning (VaDEAL) as a human-centric computing method to improve the accuracy of diagnosing pneumonia. Because active learning (AL) realizes label-efficient learning by labeling the most valuable queries, we propose a new AL strategy that incorporates clustering to improve the sampling quality. Our framework consists of a VaDE module, a task learner, and a sampling calculator. First, the VaDE performs unsupervised reduction and clustering of dimension over the entire data set. The end-to-end task learner obtains the embedding representations of the VaDE-processed sample while training the target classifier of the model. The sampling calculator will calculate the representativeness of the samples by VaDE, the uncertainty of the samples through task learning, and ensure the overall diversity of the samples by calculating the similarity constraints between the current and previous samples. With our novel design, the combination of uncertainty, representativeness, and diversity scores allows us to select the most informative samples for labeling, thus improving overall performance. With extensive experiments and evaluations performed on a large dataset, we demonstrate that our proposed method is superior to the state-of-the-art methods and has the highest accuracy in the diagnosis of pneumonia.

## 1. Introduction

COVID-19 has become a worldwide pandemic since 2019. It has infected more than 600 million population around 216 countries, with over 6.5 million death cases. In the current COVID-19 pandemic, there is an urgent need to screen infected patients quickly and accurately (Jadon, 2021). Many studies showed that deep learning models trained on chest X-ray images could become an efficient method for screening patients with COVID-19 during this pandemic. Alike to the human brain which increases the weight of the links between the nerves through frequent training (Miller and Cohen, 2001), deep learning models also need large amount of trainings. Alyasseri et al. (2022) reviewed the existing work and concluded that most of the work on COVID diagnosis were mainly based on SVM and CNN. While deep learning has achieved unprecedented success in image processing, it is not the case in CT-scan diagnosis. The disadvantage of deep learning is that it relies heavily on a large amount of labeled data, which, in most cases, is costly to obtain. Especially in the field of medical diagnosis, due to the limitation of patient numbers and their privacy, the number of labeled data is narrowed to hundreds and is insufficient for deep learning. Under these circumstances, the active learning (AL) paradigm is proposed to actively find the most informative and valuable samples for model training, hence realizing label-efficient learning and alleviating the reliability of label annotation. Concretely, AL iteratively selects the most informative samples from the unlabeled data pool to be labeled by an oracle (i.e., a human annotator) and then adds it to the labeled pool for task learner training. AL proved to be promising in various computer vision tasks (Li and Guo, 2013; Yang et al., 2017; Beluch et al., 2018; Kuo et al., 2018; Mahapatra et al., 2018).

Essentially, the main idea of AL is to design an effective sampling strategy to query the most valuable samples for the improvement of the model to get a labeled subset. The objective is to make the model trained on this subset have comparable performance to the model trained on the whole data set. Most earlier methods were derived from various task-aware ideas, such as classifier uncertainty (Tong and Koller, 2001) and expected error reduction (Yoo and Kweon, 2019). However, they were considered to be significantly affected by the scale and quality of the initially labeled data (Kim et al., 2021). A series of recent literature (Mottaghi and Yeung, 2019; Sinha et al., 2019; Zhang et al., 2020; Kim et al., 2021) have proposed adversarial AL methods, which generally use the label state information to train a discriminator to distinguish the unlabeled data representations. Adversarial methods have stronger robustness to outliers, noisy labels, and biased initial labeled data. However, they merely focus on the uncertainty of unlabeled samples, lacking a measure of other useful information, such as representativeness and diversity. In this paper, we designed our novel AL framework based on some ideas from pool-based methods. Pool-based methods are often classified into three broad categories: distribution-based, uncertainty-based, and combination. The distribution-based approach aims at selecting data that increase the diversity of the labeled pool. Those methods are proposed based on the data representation learning framework (Huang et al., 2018). In Nguyen and Smeulders (2004), diversity is improved by clustering unsupervised data and selecting samples from different clusters. In the literature on deep active learning, distribution diversity is estimated by observing gradients (Settles et al., 2007) or changes in the output of training models (Freytag et al., 2014). Uncertainty-based methods estimate the uncertainty of unlabeled data and sample the top-K most uncertain data points in each iteration. Most uncertainty-based methods derive sampling strategies from task learning. For example, these methods usually select the points with the smallest distance from the decision boundary (or classification hyperplane) (Tong and Koller, 2001) or with high information entropy (MacKay, 1992). Methods combining uncertainty and distribution consider both the uncertainty of data points and sampling diversity. Some studies have introduced the idea of adversarial representation learning into AL to learn an adversarial representation and select data points based on the discrimination for data representation. Typically, variational adversarial active learning (VAAL) (Sinha et al., 2019) learns a unified adversarial representation for labeled and unlabeled data by leveraging a generative model and estimating sample uncertainty. The learning process of VAAL is based on the difference in distribution, which is learned by the discriminator. Our proposed framework, in this study, variational deep embedding-based active learning for pneumonia diagnosis (VaDEAL), is based on the idea of adversarial representation learning, but the key difference is that in VaDEAL, the uncertainty estimate is not given by the discriminator. By introducing clustering into this adversarial architecture, our method comprehensively measures sample uncertainty, representativeness, and diversity.

In this paper, we present our framework, VaDEAL, based on the idea of human-centric computing. We remodeled the existing AL framework by adding a few more parameters for decision-making to alleviate the bias in the sampling phase and make our framework more trustworthy and fairness-aware. VaDEAL consists of three parts: a VaDE module, a task learner, and a sampling calculator. First, the VaDE performs unsupervised reduction and clustering of dimension over the entire dataset. The end-to-end task learner obtains the embedding representations of the VaDE-processed samples while training the target classifier of the model. In detail, the VaDE performs adversarial training by learning a latent space to map the data to a unified representation. The task learners are trained on latent data representations and output uncertainty estimates for sampling. The sampling calculator will calculate three scores for each sample:

1. The uncertainty score *h* is obtained from the task learner. The higher the predicted entropy of the sample, the more uncertain the sample is;
2. The representation score *G* is calculated based on clustering results. The sample at the center of the cluster is the most representative;
3. The diversity score *R* represents the similarity constraint between the current and previous samples and guarantees the overall diversity.

Finally, our AL strategy selects the sample with the largest weighted sum of uncertainty, representativeness, and diversity scores. Then, our VaDEAL framework can consider all three parameters during sampling and excel in its performance.

The contributions of this paper are summarized as follows:

- We use a VaDE algorithm to implement unified latent data representation and clustering in AL. With this, we can obtain the embedding representations of the samples for uncertainty score *h* and prevent overfitting due to the size of annotated samples being too small.
- We improve existing sampling strategies by efficiently integrating representativeness, diversity, and uncertainty, thus incorporating more information in selecting valuable unlabeled data.
- We applied this novel AL framework design to the practice of identifying pneumonia CT pictures, verifying the effectiveness of the proposed VaDEAL algorithm on a large dataset, and demonstrating that VaDEAL outperforms other state-of-art methods in diagnosing pneumonia.

## 2. Methods

**Figure 1:**
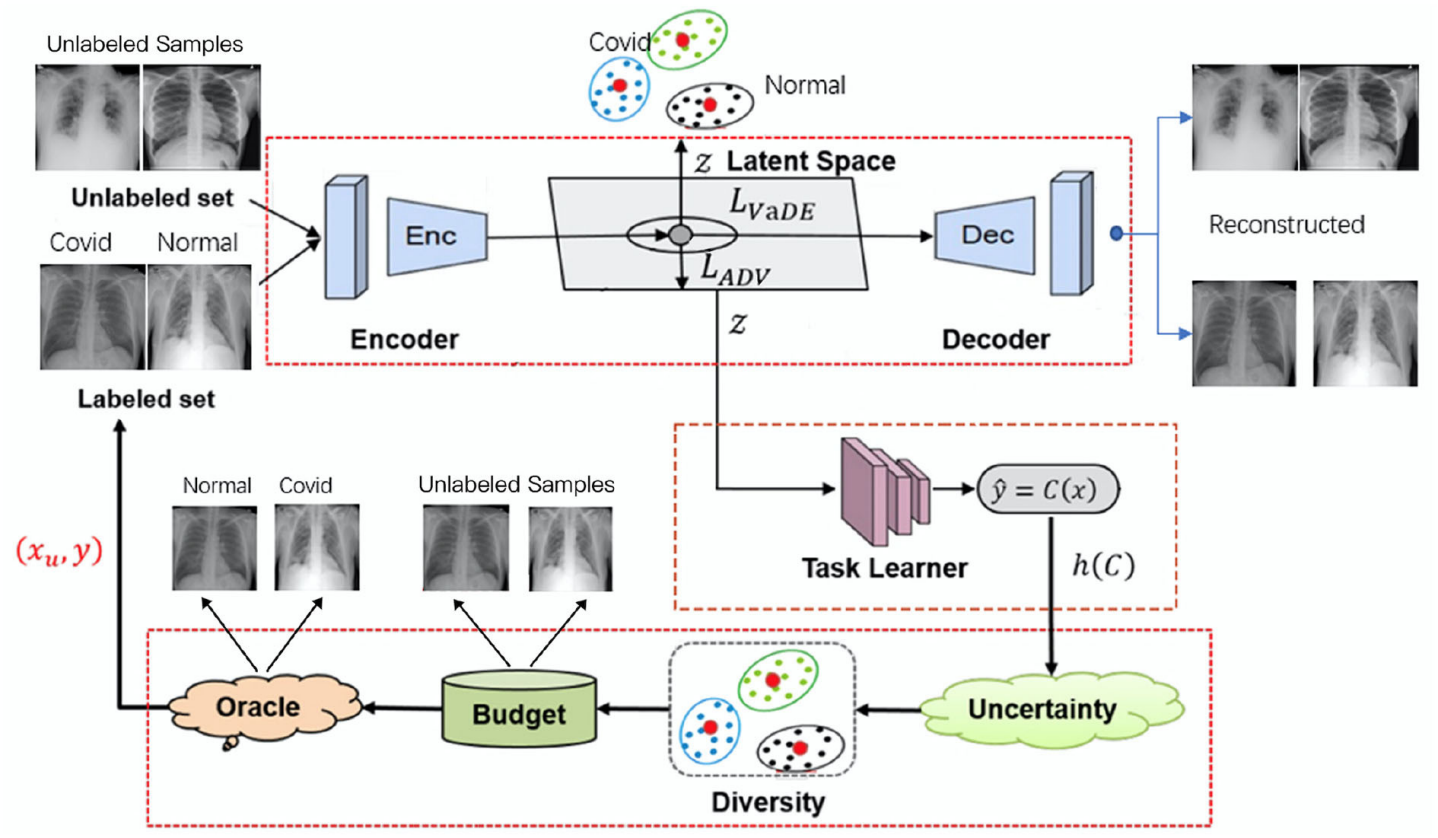
The overall framework for variational deep embedding based active learning for pneumonia diagnosis (VaDEAL).

In this section, we discuss VaDEAL framework in detail. The framework consists of three parts: a VaDE module, a task learner, and a sampling calculator. First, all the given samples are passed into the VaDE module, where samples will be reduced in dimension and clustered. Then, the samples processed by VaDE are used to train the task learner to give the uncertainty score. Finally, the sampling calculator will consider the representativeness *G* provided by VaDE, the uncertainty *h* from the task learner, and the overall diversity *R* to determine the valuable data points for labeling from the unlabeled sample pool. Therefore, our model can minimize labeling costs and build a high-performance task model. In the following paragraph, the unlabeled pool will be denoted as *X*_*U*_ and the labeled pool as *D*_*L*_. *x*_*U*_ represents a data point in the unlabeled pool, and (*x*_*L*_, *y*_*L*_) represent a data point and its annotation, respectively, in the labeled pool. The overall framework of our proposed AL algorithms is shown in Figure 1.

### 2.1. Variational deep embedding for clustering

First, we learned from the VaDE model (Jiang et al., 2016) to introduce clustering into adversarial AL. For clustering, VaDE uses GMM to pick up a cluster *c* and sample a latent representation *z* and then uses a deep neural network (DNN) *f* to decode *z* to an observation *x*. VaDE uses another DNN *g* to encode observed data *x* into latent embedding *z* to use stochastic gradient variational Bayes (SGVB) estimator (Tjandra et al., 2016) and the reparameterization trick to maximize the evidence lower bound (ELBO) $L_{ELBO}$:


(1) $$\begin{array}{l} {ℒ}_{ELBO}(x)=E_{q(z,c|x)}\log\frac{p(x,z,c)}{q(z,c|x)}\\ \;=\int_{z}q(z|x)\log\frac{p(x|z)p(z)}{q(z|x)}d_{z}\\ \;-\int_{z}q(z|x)D_{KL}(q(z|x)||p(c|z)))d_{z} \end{array}$$

where *p*(*x, z, c*) represents the joint probability which can be factorized as *p*(*x*|*z*)*p*(*z*|*c*)*p*(*c*). *q*(*z, c*|*x*) is the variational posterior to approximate the true posterior *p*(*z, c*|*x*). To maximize $L_{ELBO}$, *D*_*KL*_(*q*(*z*|*x*)||*p*(*c*|*z*)))≡0 should be satisfied.

Once the training is done by maximizing the ELBO, we can extract a latent representation for each observed sample and obtain the clustering assignments. In our sampling strategy, the clustering results are used to estimate the representativeness of the samples.

In this paper, the implementation of VaDE has two main contributions to the overall model: 1) VaDE prevents over-fitting caused by too small labeled samples, and 2) It provides clustering results to assist in sampling, from which the representative score of the sample can be obtained.

### 2.2. Task learner

The task learner obtains data representations in the VaDE latent space as input and outputs an uncertainty estimate for samples. The task learner also has two main functions: 1) to get the classification result and 2) to provide the uncertainty score of the sample. The loss function of the task leaner is


(2) $$\begin{array}{l} \text{Loss}=-\overset{\beta }{\underset{x}{\sum }}\overset{C-1}{\underset{i=0}{\sum }}q\left(y=i|x\right)\log p\left(y=i|x\right) \end{array}$$

where *q* is the actual label probability of *x*, when *x* is from a labeled pool. β represents the total number of samples, and *p* represents the probability that the model predicts on *x*. The classification uncertainty of the sample will be


(3) $$\begin{array}{l} h{\left(x\right)}_{c}=\overset{L}{\underset{i}{\sum }}p\left(y=i|x\right)\log\;p\left(y=i|x\right) \end{array}$$

where *L* is the number of Monte Carlo samples in the SGVB estimator.

### 2.3. Sampling calculator

The sampling calculator will calculate the weighted sum of the three scores for each sample:

Both representativeness and diversity can be optimized from clustering, but we still calculate them separately to give a specific metric.

1. **Representativeness**: Data points are clustered and the similarity between data points are computed within the cluster. First, the similarity of the current sample to other samples in the same cluster is calculated, and then averaged (the samples in the center of the cluster are the most representative).
2. **Uncertainty**: *h*(*x*)_*c*_ from Equation (3) will be served as the uncertainty score in the sampling calculator (the higher the predicted entropy, the more uncertain the sample).
3. **Diversity**: Diversity is related to the sampled set *S*. For each unlabeled sample point, the similarity is calculated with all samples in the *S*, then averaged, and the negative is taken.

With the framework given above, the sampling strategy of the whole model is as follows:

1. First, the uncertainty *H* and representative *G* of all unlabeled samples are calculated.
2. The diversity *R* is calculated for each sample of the sampled set *S* (if S is empty, the diversity can be set to 0).
3. The composite score is calculated for the sample *I* = *H*+r*G+b*R, where *r* and *b* are the weights of representative and diversity, respectively. *r, b* will be chosen within the scope 0.1, 0.5, 1, and 5, and each combination will be tried for the best performance.
4. The sample *I* is sorted in descending order, for all the samples outside *S*, find the one with the largest score *I* and put it into *S*.
5. The above process is repeated until the size of *S* is equal to budget *B*, the sample size for each round of sampling.

Therefore, our model considers all three important criteria of sampling, namely uncertainty, representativeness, and diversity, which current AL community emphasizes.

## 3. Experiment

**Dataset**. We used the ChestX-ray14 dataset provided by the NIH Research Institute (Wang et al., 2017), which is the largest chest X-ray image dataset with the most disease types. The ChestX-ray14 dataset contains 14 chest diseases and normal samples where no infection was found. This paper only considered single-label samples, as well as normal samples, for 15 categories with 21000 X-ray PNG images in 1024 × 1024 resolution.

**Baselines**. We compared our method with the recent state-of-the-art AL approaches:

1. **Random sampling** Vitter (1985) uniformly picks the samples from the unlabeled pool, and then train the classifier on the labeled data.
2. **Max-entropy** Wang and Shang (2014) picks unlabeled instances with the highest entropy.
3. **Feature mixing** Parvaneh et al. (2022) identifies informative unlabeled instances by evaluating the variability of the labels predicted for perturbed versions of these instances and explores the neighborhood surrounding an unlabeled instance by interpolating its features with those of previously-labeled ones.

**Implementation detail**. We initialized the labeled pool *D*_*L*_ by randomly selecting a part of the whole dataset and the unlabeled data *D*_*U*_ with the remaining data. The AL program selected and labeled the samples from *D*_*U*_ in the experiments. The newly labeled samples were appended to *D*_*L*_, and the training was repeated on the new *D*_*L*_. We measured the accuracy of the classifier trained in the AL process as *D*_*L*_ grew. We computed the average accuracy of five experiments for the reliability of the experiment. The data (*D*_*L*_, *D*_*U*_) of each experiment were randomly initialized. The 5-layer multilayer perceptron (MLP) was used as a task learner, and the VaDE we used in this study has the same architecture and hyperparameters setting as the VaDE in the literature (Jiang et al., 2016). The parameters of SDG were set as 0.9 momentum and 0.005 weight decay. The learning rate for the task learner was 1*e*^−4^. The batch size of all training modules was 64, and the sample size of each AL round was 100.

**Results**. We then compared the performances by evaluating micro-F1, ACC, and AUC scores. From Tables 1, 2, the performance of three baseline methods and our framework improved after each active learning rounds. After five rounds, the feature-mixing strategy has the best performance among the three baseline methods, as highlighted in Table 1. The performance of our framework after five rounds with a percentage improvement compared to feature-mixing was also highlighted in Table 2. Compared to three baselines, our proposed framework outperformed all three baseline methods under any AL round, with 27.61, 15.03, and 11.41% improvements in the F1 score compared to random-sampling strategy, maximum-entropy, and feature-mixing, respectively.

**Table 1:** Different sampling strategies.

**Strategy**	**Random**			**Max-entropy**			**Feature-mixing**		
**AL rounds**	**ACC**	**micro-F1**	**AVG-AUC**	**ACC**	**micro-F1**	**AVG-AUC**	**ACC**	**micro-F1**	**AVG-AUC**
0	0.5982	0.5162	0.7322	0.5982	0.5162	0.7322	0.5982	0.5162	0.7322
1	0.6464	0.5645	0.7642	0.6678	0.6196	0.7785	0.6773	0.6134	0.7848
2	0.6738	0.6079	0.7825	0.6884	0.6538	0.7923	0.6961	0.6621	0.7974
3	0.6944	0.6314	0.7963	0.7064	0.6809	0.8043	0.7185	0.6870	0.8123
4	0.7090	0.6345	0.8060	0.7253	0.7006	0.8169	0.7305	0.7081	0.8203
5	0.6858	0.6352	0.7906	0.7313	0.7047	0.8209	**0.7511**	**0.7276**	**0.8340**

**Table 2:** Variational deep embedding based active learning for pneumonia diagnosis (VaDEAL) framework.

**AL rounds**	**0**	**1**	**2**	**3**	**4**	**5**	**Over best of baselines after 5 rounds**
ACC	0.7474	0.7842	0.8193	0.8126	0.8291	**0.8356**	**11.25%**
micro-F1	0.6916	0.7450	0.7761	0.7642	0.8037	**0.8106**	**11.41%**
AVG-AUC	0.9185	0.9421	0.9467	0.9456	0.9515	**0.9530**	**14.27%**

## 4. Discussion

In this paper, we proposed a new AL algorithm VaDEAL, that fully used label information and hidden clustering information to improve the sampling quality in AL. VaDEAL is a computer-automatic diagnosis approach that aims to improve the accuracy of the diagnosis of pneumonia. It is the human-centric computing (HCC) approach that human beings are treated only at an end. VaDEAL is designed to be more trustworthy and fairness-aware as we remodeled the framework to consider more label information to alleviate the bias during sampling. It performs brain-like computing by adjusting the parameters on the training set to achieve the classification task on the diagnosis of pneumonia. We build our framework with three modules:

- A VaDE module that implements unsupervised clustering on the samples and also helps to obtain samples' representation score *G*;
- A task learner is trained to get the classification result and provide the uncertainty score *h* of samples;
- A sampling calculator adds the weighted sum of *R, G*, and *h* scores to determine the value of each sample so that our framework can comprehensively consider all three scores and fully use the label information and hidden clustering pattern in data.

We implemented our novel framework in diagnoses of pneumonia, and with extensive experiments on the ChestX-ray14 dataset, we demonstrated that VaDEAL outperformed all three baseline methods and showed a larger potential.

## Data availability statement

Publicly available datasets were analyzed in this study. This data can be found at: https://openaccess.thecvf.com/content_cvpr_2017/papers/Wang_ChestX-ray8_Hospital-Scale_Chest_CVPR_2017_paper.

## Author contributions

JH and GY made great contributions on proposing and designing the integration of AI and medicine. WD primarily focused on the AI research and applications concerning pneumonia diagnosis. JZ investigated and summarized the reviewed studies using active learning framework and wrote and revised articles with the other co-authors. ZL and TS was primarily responsible for collecting related research and preparing scrub data. All authors contributed to the article and approved the submitted version.

## Funding

This work was partially supported by the National Key R&D Program of China (No. 2019YFE0126200) and the National Natural Science Foundation of China (No. 62076218).

## Conflict of interest

Author PK was employed by Avaintec Oy. Author GZ was employed by JancsiTech. The remaining authors declare that the research was conducted in the absence of any commercial or financial relationships that could be construed as a potential conflict of interest.

## Publisher's note

All claims expressed in this article are solely those of the authors and do not necessarily represent those of their affiliated organizations, or those of the publisher, the editors and the reviewers. Any product that may be evaluated in this article, or claim that may be made by its manufacturer, is not guaranteed or endorsed by the publisher.
